# Supplementary material for: Acceptance and compliance with micronutrient powder (MNP) among children aged 6–23 months in northern Nigeria
Source: PLOS Glob Public Health. 2022 Oct 17;2(10):e0000961. doi: 10.1371/journal.pgph.0000961 (PMC10022258; doi:10.1371/journal.pgph.0000961)
Supplement: S1 Dataset — (ZIP) [file pgph.0000961.s008.zip › S8_Dataset/Direct Observations.zip!/Direct Observations/07072015_A_GA_AS_2_DO.pdf]

1.1.1 Instrument F –Direct Observation Form

<<Instrument F Direct Observation Form>>
**PI Name:** PragyaMathema,Stephen Kodish<br>**Study Title:** Formative research to develop an infant and young child feeding (IYCF) intervention with micronutrient powder (MNP) for children aged 6 – 23 months<br>**PI Version/Date:** Version1.0/January 24, 2015

Date: _July 07, 2015____________Location: _Ganye 1 community, LGA -Ganye

Data Collector: Antiya Sanda Participant ID: A house near a well

Main goals of this direct observation are to answer the following questions:
1. In this household, what would be the best way(s) to give nutritional supplements to young children? Why?<br><br>2. In this household, describe the food sharing that you observe throughout the meal. Are there any predictable patterns?<br><br>3. In this household, who is favored and how is this favoritism expressed during meal preparation and eating?

(TEAR OFF AT PERFORATED DOTTED LINE BELOW)

PARTICIPANT ID: __

Household Composition Table (Fill out as you do data collection)

INITIALS	SEX (M/F)	AGE (estimated)	Relationship to household	Description
TB	F	30 YRS	Mother	A tall black woman wearing a yellow.
SB	F	7 MONTHS	Child (observed child)	A small black baby.
TP	M	35 Yrs	Father	A tall man wearing a purple shirt an ash black blouse.
AB	F	28 Yrs	daughter	A small baby wearing a purple dress
SP	F	2 Yrs	Daughter	A little boy wearing a while and brown shorts
WB	M	3 Yrs		

PARTICIPANT ID: __________

TIME	ACTOR INITIALS	RECIPIENT INITIALS	WHAT HAPPENED	FOOD	Reactivity
8.00	TB		Sweeping the house		
8:05	TB	SB	Carry her child on the back		
8:10	TB		Sweeping the house		
8:15	TB		Sweeping		
8:20	TB		Sweeping		
8:25	TB		Brings out some plates to wash		
8:30	TB		Fetching water		
8:35	TB		Washing plates		
8:40	TB		Washing the plates		
8:45	TB		Rinsing plates		
8:50	TB		Rinsing the plates		
8:55	TB		Rinsing the plates		
9:00	TB	SB	Breastfeeding	Breast milk	
9:05	TB	SB	Breastfeeding	Breast milk	
9:10	TB	SB	Breastfeeding	Breast milk	
9:15	TB	SB	Breastfeeding	Breast milk	
9:20	TB	SB	Breastfeeding	Breast	

TIME	ACTOR INITIALS	RECIPIENT INITIALS	WHAT HAPPENED	FOOD	Reactivity
				milk	
9:25	TB	SB	Carry her child on the back gives into room and bring baby's dirty clothes		
9:30	TB		Goes into the room and bring baby dirty clothes		
9:35	TB		Pour water into a big bucket		
9:40	TB		Bring detergent		
9:45	TB		Start washing the baby clothes		
9:50	TB		Washing some clothes		
9:55	TB		Washing some clothes		
10:00	TB		Washing some clothes		
10:05	TB		Start rinsing the clothes		
10:10	TB		Rinsing the baby clothes		
10:15	TB		Rinsing the baby clothes		
10:20	TB		Spread clothes for drying		
10:25	TB		Drying baby clothes		
10:30	TB		Drying baby's clothes		
10:35	TB		Pouring the dirty water outside		
10:40	TB		Bring back the bucket to keep		
10:45	TB	SB	Goes into the room to lay her child		
10:50	SB		Sleeping		
10:55	SB		Sleeping		
11:00	SB		Sleeping		
11:05	SB		Sleeping		
11:10	SB		Sleeping		
11:15	SB		Sleeping		
11:20	SB		Sleeping		
11:25	SB		Sleeping		
11:30	SB		Sleeping		
11:35	SB		Sleeping		
11:40	SB		Sleeping		
11:45	SB		Sleeping		
11:50	TB		Fetches water into a bucket to		

TIME	ACTOR INITIALS	RECIPIENT INITIALS	WHAT HAPPENED	FOOD	Reactivity
			bath		
11:55	TB		Bathing		
12:00	TB		Bathing		
12:05	TP		Walk into the house		
12:10	TP		Speaks with the wife and leaves		
12:15	TB		Comes out of the room well dressed		
12:20	TB		Picks a bucket and begins to wash it		
12:25	TB		Fetches water into the bucket		
12:30	TB		Start rinsing big water pot		
12:35	TB		Pick a bucket and goes to fetch water from the well		
12:40	TB		Fetching water from the well		
12:45	TB		Fetching water from the well		
12:50	TB		Fetching water from the well		
1:00	TB		Fetching water from the well		
1:05	TB		Fetching water from the well		
1:10	TB		Fetching water from the well		
1:15	TB		Start making fire		
1:20	TB		Washing fresh pepper and fresh tomatoes	Tomatoes and pepper	
1:21	TB		Bring mortar		
1:22	TB		Pour tomatoes and pepper into mortar	Tomatoes and pepper	
1:23	TB		Blowing fire		
1:24	TB		Blowing the fire	Tomato and pepper	
1:25	TB		Start pounding the tomatoes and peper	Tomatoes and pepper	

TIME	ACTOR INITIALS	RECIPIENT INITIALS	WHAT HAPPENED	FOOD	Reactivity
1:26	TB		Pounding pepper and tomatoes	Tomatoes and pepper	
1:27	TB		Pounding pepper and tomatoes	Tomatoes and pepper	
1:28	TB		Pounding pepper and tomatoes	Tomatoes and pepper	
1:29	TB		Pounding pepper and tomatoes	Tomatoes and pepper	
1:30	TB		Pounding pepper and tomatoes	Tomatoes and pepper	
1:31	TB	SB	Carry baby on her arms		
1:32	TP	SB	Collect the baby		
1:34	TB		Open the flask		
1:35	TB		Pour hot water into a small cup	Water	
1:36	TB		Bring the can of cerelac	Cerelac	
1:37	TB		Take two spoonful of cerelac into a cup	Cerelac	
1:38	TB		Mixing cerelac with hot water	Cerelac and hot water	
1:39	TB		Stirring the cerelac and hot water	Cerelac and hot water	
1:40	TB		Still stirring it to make it cold	Cerelac and water	
1:41	TB		Bring out packet of MNp	MNP	
1:42	TB		Removed one sachet	MNP	
1:43	TB		Take scissors		
1:44	TB		Cut the tip with scissors	MNP	
1:45	TB		Pour MNP into the cerelac and stirs it		
1:46	TB		Drop empty sachet of MNP		

TIME	ACTOR INITIALS	RECIPIENT INITIALS	WHAT HAPPENED	FOOD	Reactivity
1:47	TB		Stirring of MNP and cerelac	MNP, cerelac	
1:49	TB		Use a spoon to taste the mixture	MNP , cerelac	
1:50	TB	SB	Collects the baby from the father		
1:51	TB	SB	Carry the baby on the lap		
1:52	TB	SB	Wears the baby a beep		
1:53	TB	SB	Start feeding the baby using a small spoon	MNP, Cerelac	
1:54	TB		Feeding the child	MNP, cerelac	
1:55	TB		Feeding the child	MNP, cerelac	
1:56	TB		Feeding the child	MNP, cerelac	
1:57	TB		Feeding the child	MNP, cerelac	
1:58	TB		Feeding the child	MNP, cerelac	
1:59	TB		Feeding the child	MNP, cerelac	
2:00	TB		Feeding the child	MNP, cerelac	
2:01	SB		Drink all cerelac mixed with MNP		
2:02	TB		Clean the child mouth with beep		
2:05	TB	SB	Gives her child water to drink		
2:10	SB		Drinking the water slowly	Water	
2:15	Tb	SB	Carry the child on the arm		
2:20	TB		Picks a cup to fetch water		
2:25	TB		Drinking water	Water	
2:30	TB	SB	Piggy back her child		
2:31	TB		Bring a spoon		

TIME	ACTOR INITIALS	RECIPIENT INITIALS	WHAT HAPPENED	FOOD	Reactivity
2:32	TB		Use spoon to fetch the pounded tomatoes from mortar into a pot	Tomatoes and pepper	
2:34	TB		Places the pot on fire	Tomatoes and pepper	
2:35	TB		Sing hand fan to blow fire		
2:36	TB		Bring palm oil in a bottle	Palm oil	
2:37	TB		Pour palm oil inside the pot of pepper on fire	Pepper, tomatoes and palm oil	
2:38	TB		Add little water into the mortar and stirs it	Water	
2:39	TB		Pour the water from the mortar into a bowl		
2:40	TB		Adding magi star cube	Seasoning	
2:41	TB		Still unwrapping magi	Seasoning	
2:42	TB		Using hand fan to blow the fire		
2:43	TB		Uses a spoon to stir the pepper and and tomatoes on the fire	Pepper and tomatoes, palm oil	
2:44	TB		Sit and wait for it to fry		
2:45	TB		Stirring		
2:46	TB		Covers the pot		
2:47	TB		Blowing fire		
2:48	TB		Re arranging firewood		
2:49	TB		Opens pot and add little water	Water	
2:50	TB		Cover pot		
2:51	TB		Allow the stew to cook	Tomaoes and pepper	
2:52	TB		Blow fire		
2:53	TB		Blow fire		
2:54	TB		Open the pot and leave it open		
2:55	TB		Goes into the kitchen and spring	Spaghetti	

TIME	ACTOR INITIALS	RECIPIENT INITIALS	WHAT HAPPENED	FOOD	Reactivity
			out spaghetti		
2:56	TB		Open the packet of spaghetti		
2:57	TB		Break the strands into pieces	Spaghetti	
2:58	TB		Pour spaghetti into the pot	Spaghetti	
2:59	TB		Use spoon to stir the spaghetti	Spaghetti	
3:00	TB		Drop the spoon on the plate		
3:01	TB		Cover the pot		
3:03	TB		Fetch water to drink with a cup	Water	
3:04	Tb		Drink water		
3:05	TB		Open the pot on the fire		
3:06	TB		Stirring spaghetti on fire	Spaghetti	
3:07	TB		Leaves the pot open		
3:08	TB		Add little salt	Salt	
3:09	TB		Add little water		
3:10	TB		Cover the pot		
3:11	TB		Start sweeping the kitchen		
3:12	TB		Sweeping the kitchen		
3:13	TB		Pick a rag to open the pot on the fire		
3:14	TB		Bring down the pot of spaghetti from fire		
3:15	TB		Puts another pot on the fire		
3:16	TB		Takes hot pot to the kitchen		
3:17	TB		Pick food warmer		
3:18	TB		Serves food	spaghetti	
3:19	TP		Walk into the house		
3:20	TB	TP	Speaking with the wife		
3:21	TB		Continues to serve the spaghetti	Spaghett i	
3:22	TB		Serving the spaghetti	Spaghett i	
3:23	TB		Stop serving the spaghetti		
3:24	TB		Covering the food warmer and the plates		
3:25	TB		Pack all the plates into the room		
3:30	TP		Walk into the house		
3:35	SB		Sleeping		

TIME	ACTOR INITIALS	RECIPIENT INITIALS	WHAT HAPPENED	FOOD	Reactivity
3:40	TB		Bring the food warmer out with a tray		
3:45	TB		Bring a flat plate and start serving the spaghetti	Spaghett i	
3:50	TB		Bring a spoon and water		
3:55	TB,TP		Eating food together	Spaghett i	
4:00	TB,TP		Eating together	Spaghett i	
4:05	TB,TP		Eating together	Spaghett i	
4:10	TB,TP		Eating together	Spaghett i	
4:15	TB		Drinking water	Water	
4:20	TP		Drinking water	Water	
4:25	TB,TP		Eating together	Spaghett i	
4:30	TB,TP		Eating together	Spaghett i	
4:35	TB,TP		Eating together	Spaghett i	
4:40	TB,TP		Eating together	Spaghett i	
4:45	TP		Walk out of the house		
4:50	TB		Start packing all the plates to the kitchen		
4:55	TB		Clearing the plates		
5:00	SB		Crying from the room		
5:05	TB	SB	Carry her child on the lap		
5:10	TB		Uses ordinary water to wash hands		
5:15	TB	SB	Breastfeeding	Breast milk	
5:20	TB	SB	Breastfeeding	Breast milk	
5:25	TB	SB	Breastfeeding	Breast milk	
5:30	TB	SB	Breastfeeding	Breast milk	

TIME	ACTOR INITIALS	RECIPIENT INITIALS	WHAT HAPPENED	FOOD	Reactivity
5:35	TB	SB	Breastfeeding	Breast milk	
5:40	TB	SB	Breastfeeding	Breast milk	
5:45	TB	SB	Breastfeeding	Breast milk	
5:50	TB	SB	Breastfeeding	Breast milk	
5:55	TB	SB	Breastfeeding	Breast milk	
6:00	TB	SB	Breastfeeding	Breast milk	

PARTICIPANT ID: __ _______

Primary Questions (Complete after you complete data collection):

From what you have seen observing THIS HOUSEHOLD, please answer the following questions in detail:

Question	Write your detailed response here
1. In this household, what would be the best way(s) to give nutritional supplements such as an MNP to young children to ensure compliance?<br><br>Why?	The best way and time to give the MNP should be in the afternoon because that is when children are fed with semi solid food and the child is already used to the system.<br><br>Because this young child sleeps throughout in the morning and wakes up hungry in the afternoon.
2. In this household, who is favored during meal preparation or feeding?<br><br>How is this favoritism expressed?	The male and female caregivers are favoured during meal preparation.<br><br>They are served the first and foremost before anyone else. They also served the largest portion in a nice and good food warmer.
3. How could we promote the MNP supplement for this	MNP can be promoted through the male and female caregiver.

family that would attract their attention?<br><br>Who would they listen to?	The young child would listen to both the father and the mother, because he is used to having both parents at home at all time.
4. In this household, what would be the best ways to promote the MNP but limit how much it would be shared?<br><br>Why?	The best way would be through the mother and father. The MNP may likely not be shared because this parents are educated and can follow the instructions stated on the pamphlet.<br><br>The parents of this young child already know the basic of MNP and the usage , the tendency that it would be shared is minimal.
5. What other issues and concerns did you see with this family that are important for us to consider with regard to introduction of an MNP?<br><br>Why?	There no concern or issues as far as this household is concern.<br><br>The parents of this child work hand in hand and already knows how to maintain good hygiene for the child. The food availability in this household is stable.
6. Based on your experience observing this child today, do you believe reactivity negatively impacted your ability to see the food-related behaviors of the child?<br><br>Please explain your answer in detail.	Reactivity did not affect my stay and observation process.<br><br>There was no such thing like reactivity today. Because the parents are already used to having me around all day.
